# Multimorbidity in Focus: Emerging Patterns, Medication Appropriateness and Clinical Innovation

**DOI:** 10.3390/jcm15010101

**Published:** 2025-12-23

**Authors:** Ignatios Ioakeim-Skoufa, Mercedes Aza-Pascual-Salcedo, Helena Coelho, Jorge Vicente-Romero

**Affiliations:** 1 Department of Drug Statistics, Division of Health Data and Digitalisation, Norwegian Institute of Public Health, 0213 Oslo, Norway; 2 Department of Pharmacology, Physiology, and Legal and Forensic Medicine, Faculty of Medicine, University of Zaragoza, 50009 Zaragoza, Spain; 3 EpiChron Research Group on Chronic Diseases, Aragon Health Research Institute (IIS Aragón), Miguel Servet University Hospital, 50009 Zaragoza, Spain; 4 Drug Utilisation Work Group, Spanish Society of Family and Community Medicine (semFYC), 08009 Barcelona, Spain; 5 Research Network on Chronicity, Primary Care, and Health Promotion (RICAPPS), Institute of Health Carlos III (ISCIII), 28029 Madrid, Spain; 6 Research Network on Global Evidence on Drug Regulation and Safety (GEDRAS), 50009 Zaragoza, Spain; 7 Primary Care Pharmacy Service Zaragoza III, Aragon Health Service (SALUD), 50017 Zaragoza, Spain; 8 Portuguese Society of Health Care Pharmacists (SPFCS), 3000-316 Coimbra, Portugal

Chronic conditions and multimorbidity are significantly altering the landscape of modern clinical care. When we launched this Special Issue, our goal was to compile research that sheds light on these challenges from various perspectives, including clinical, epidemiological, methodological and patient-centred. This Special Issue provides insights into disease trajectories, medication safety, vulnerable populations and emerging tools to understand and address multimorbidity, such as artificial intelligence (AI). We are grateful to all the contributing authors, the reviewers who enhanced the manuscripts and the editorial team of the *Journal of Clinical Medicine* for their support in this endeavour.

Recent advancements in medicine and technology have improved the survival and management of chronic conditions. Multimorbidity has now become a common clinical concern among adults of all ages, from the young population to people over 100 [1,2,3,4]. This epidemiological shift poses significant challenges for healthcare providers and national health and social care systems, where clinical guidelines mainly focus on single diseases, failing to consider a person-centred approach in the management of people with multiple chronic conditions. The contributions in this Special Issue collectively underline that multimorbidity is not a static construct but a dynamic process that evolves across life stages, involves clinical and pharmacological interactions, and requires a re-engineering of current models of care.

## 1. Clinical Trajectories

A systematic review on trajectories of cardiometabolic multimorbidity shows that cardiometabolic conditions frequently act as central nodes in the evolution of multimorbidity [5]. Hypertension, dyslipidaemia and diabetes mellitus often evolve towards more complex patterns with additional cardiovascular diseases, renal conditions, mental health problems and functional decline. Importantly, these transitions are not confined to older adults but already appear in early mid-life. Since many cardiovascular and metabolic conditions are partly preventable and linked to modifiable lifestyle factors, these findings call for the implementation of effective strategies targeted at younger populations.

The review on the use of electronic health records and AI extends this idea by demonstrating how machine-learning models, from tree-based approaches to deep learning, are increasingly used to identify complex clinical profiles, predict progression to higher morbidity burden and anticipate adverse outcomes in patients with multimorbidity [6]. The review highlights both opportunities and challenges in this domain: while AI enables the analysis of combinations of conditions beyond traditional clinical reasoning, issues of external validation, heterogeneity in outcome definitions and inconsistent reporting remain major obstacles.

## 2. Medication Safety

A systematic review of interventions to address polypharmacy in Italy identified only nine studies (seven observational, two experimental) that met standards for evaluating interventions to monitor or manage potentially inappropriate polypharmacy [7]. Results show interventions are sparse and unevenly implemented, with monitoring being the most common approach of intervention. Outcomes assessed included reductions in the number of medications, changes in comorbidity burden, measures of effectiveness, and some cost-saving, but the heterogeneity of methods and scant quantity of data made clear conclusions elusive.

Two original articles focus on prescribing appropriateness and drug-related problems (DRPs) in older adults, providing a detailed look into hospital and emergency environments.

In a tertiary hospital study, Pramotesiri and colleagues report that many patients were exposed to potentially inappropriate medication, harmful drug–drug interactions and avoidable adverse drug events [8]. Proton-pump inhibitors and cancer therapies emerged as frequent sources of concern, reflecting prescribing habits that may not always be adapted to frail, clinically complex patients. The analysis shows that multimorbidity and polypharmacy reinforce one another, increasing vulnerability to errors, suboptimal treatment choices and medication burden, especially when medications include proton-pump inhibitors, hypoglycemics, diuretics, psychotropics or cardiovascular drugs. The authors argue for structured medication reviews, closer clinical oversight and more deliberate prescribing practices.

Brannigan and colleagues extend this picture to emergency admissions [9]. They investigate the relationship between potentially inappropriate prescribing, prescribing omissions, and hospital admissions related to adverse drug reactions among older adults admitted acutely to the hospital. Using updated STOPP/START and Beers Criteria, the authors found that patients with potentially inappropriate prescribing and prescribing omissions were more likely to be admitted due to adverse drug reactions than those with more appropriate prescribing patterns. The findings suggest that in older, multimorbid individuals, both overprescribing and underprescribing carry risks. The authors highlight the need for careful medication review and optimisation in chronic, complex patients to reduce avoidable harm and support the safer management of multimorbidity.

The analysis of drug–drug interactions using EudraVigilance provides further insight into how safety considerations should be refined [10]. The study explores real-life risks of drug–drug interactions involving selective serotonin reuptake inhibitors (SSRIs). The authors analyse spontaneous safety reports and show that when SSRIs are combined with other antidepressants or psychotropic drugs, the likelihood of harmful interactions rises. The findings underscore the importance of regular medication review in people with multiple chronic condition.

## 3. Younger and Vulnerable Populations

A descriptive study conducted in Norway explores morbidity patterns among adolescents prescribed hypnotic drugs [11]. Most users had psychiatric comorbidity, including behavioural, emotional and neurodevelopmental disorders, while only a small minority had a diagnosis of sleep disorder. Alongside these mental health conditions, many also lived with somatic comorbidities such as asthma, musculoskeletal problems and headaches, reflecting a broader pattern of chronic and overlapping health needs. The authors suggest that hypnotics may often be used as a response to distress linked to complex morbidity rather than to insomnia itself. This raises concerns about diagnostic precision, continuity of care and the potential for inappropriate pharmacological management. The authors also highlight the need for a more comprehensive assessment and non-pharmacological approaches.

In a different setting, an observational study in South Africa revealed that, among people with substance use disorder, patients who also meet criteria for attention-deficit/hyperactivity disorder (ADHD) tend to show more severe depressive symptoms than those without ADHD, especially men [12]. The authors emphasise the need for integrated psychiatric assessment and management within addiction services. They also illustrate how multimorbidity in younger or socially marginalised populations frequently involves mental health clusters that require dedicated models of care.

## 4. Patient Experience and Beliefs

Managing chronicity also requires attention to how patients perceive their treatments. Muñoz-Cobos and colleagues validated a short questionnaire designed to assess beliefs about inhaled therapy in patients with Chronic Obstructive Pulmonary Disease (COPD), a chronic disease often accompanied by other long-term conditions [13]. The authors show the instrument is feasible in primary-care settings and yields acceptable psychometric reliability. Importantly, when applied to a real-world cohort of COPD patients, many of whom carry substantial comorbidity and thus complex medication regimens, the questionnaire revealed substantial levels of non-adherence to inhaled therapy. Given that adequate inhaled treatment is central to COPD management and that poor adherence may worsen the burden of chronic disease and interact poorly with other medical therapies, this tool helps identify patients at risk. The study underlines that understanding patients’ beliefs and adherence behaviours is a valuable complement to comprehensive and person-centred care, contributing to safer and more appropriate clinical management.

## 5. A Research and Policy Agenda for the Years Ahead

The articles presented here [5,6,7,8,9,10,11,12,13], together with existing research in the field [1,2,3,14,15,16,17,18,19], point towards several areas of work that merit increase attention in the near future:

1. *Identifying early patterns in the onset of multimorbidity and long-term trajectories*

A key priority is to understand when multimorbidity begins and how its early patterns evolve over time. This requires longitudinal studies that follow individuals from childhood, adolescence and early adulthood into later life, allowing researchers to identify which combinations of conditions appear first, how they cluster and which trajectories carry the highest clinical, functional and therapeutic risks. Such knowledge is essential for designing population-level prevention strategies and for recognising, early on, the individuals most likely to develop complex multimorbidity in later years.

2. *Making real-world data and AI genuinely useful for clinicians*

Electronic health records and AI-driven models will only improve multimorbidity care if their predictions are reliable, transparent and connected to decisions that clinicians can act upon. This calls for better external validation, clearer reporting standards and a stronger link between data-driven insights and day-to-day clinical practice.

3. *Strengthening medication safety in the context of multimorbidity*

Medication safety becomes more difficult to manage as people accumulate more conditions and more treatments over the years. Approaches to medication review that consider comorbidities, expected interactions between drugs and conditions, and the priorities and circumstances of each person may allow for a more accurate understanding of risk.

4. *Redesigning care around vulnerability and complexity*

Vulnerability in multimorbidity is often limited to simple labels, such as age, number of diseases, and number of medicines. The studies in this Special Issue show that this approach is not sufficient to identify vulnerable or high-risk groups. Vulnerability emerges from the interplay of clinical instability, mental health problems, medication burden, family and social context, economic constraints, educational background and cultural attitudes towards illness and treatment. Future research should develop approaches that recognise patterns of fragility considering clinical, behavioural and sociocultural factors.

5. *Embedding patient experience in routine practice*

The long-term management of multimorbidity depends as much on clinical decisions as on how people live with their treatments. Expectations, concerns and personal priorities influence whether a plan is workable, sustainable or even understood. Recording patients’ experiences (how they feel about their medicines, what challenges they face in daily routines, and how satisfied they are with their care) can offer valuable insights. Such approaches, whether through structured tools or more open conversations, tend to reveal early signs of treatment fatigue, misunderstanding or situations where clinical plans drift away from what the person actually values or feels able to manage. Bringing this dimension into practice enriches clinical judgement and helps shape treatment plans that make sense not only medically, but also in the context of people’s lives.

6. *Harmonising definitions and outcomes in multimorbidity research*

Studies often rely on different disease lists, timeframes or classification systems, making it difficult to compare findings or translate evidence into policy and clinical practice. The field would benefit from an agreed set of core outcomes that capture not only disease counts, but also functional measures, treatment burden, medication safety and patient-reported priorities. A more unified framework would strengthen the evidence base, support replication across countries and help policymakers plan services grounded in reliable, comparable data.

If the coming years bring progress across these areas, health systems will be better positioned to meet the needs of people living with multimorbidity.

## 6. Conclusions

The articles in this Special Issue show how chronicity manifests across life stages, settings and therapeutic domains. They also show that multimorbidity is a multidimensional challenge: biological, clinical, pharmacological, and social. They demonstrate that clinical trajectories often begin earlier than assumed; medication safety requires continuous vigilance; vulnerable populations face unique and often overlooked burdens; patient beliefs shape treatment adherence; and real-world data and AI have the potential to enhance, but not replace, clinical reasoning. Real-world data and AI are becoming indispensable tools in this landscape, but their value depends on responsible implementation, transparency in methods, and careful consideration of their limitations. Recent reflections on real-world evidence in regulatory settings and policy-making highlight the need for rigour, reproducibility and clear reporting [20].

## Data Availability

Data is contained within the article.
